# Is There a Canonical Cortical Circuit for the Cholinergic System? Anatomical Differences Across Common Model Systems

**DOI:** 10.3389/fncir.2018.00008

**Published:** 2018-01-30

**Authors:** Jennifer J. Coppola, Anita A. Disney

**Affiliations:** Department of Psychology, Vanderbilt University, Nashville, TN, United States

**Keywords:** neuromodulation, acetylcholine, comparative anatomy, cortex, macaque, human, rat

## Abstract

Acetylcholine (ACh) is believed to act as a neuromodulator in cortical circuits that support cognition, specifically in processes including learning, memory consolidation, vigilance, arousal and attention. The cholinergic modulation of cortical processes is studied in many model systems including rodents, cats and primates. Further, these studies are performed in cortical areas ranging from the primary visual cortex to the prefrontal cortex and using diverse methodologies. The results of these studies have been combined into singular models of function—a practice based on an implicit assumption that the various model systems are equivalent and interchangeable. However, comparative anatomy both within and across species reveals important differences in the structure of the cholinergic system. Here, we will review anatomical data including innervation patterns, receptor expression, synthesis and release compared across species and cortical area with a focus on rodents and primates. We argue that these data suggest no canonical cortical model system exists for the cholinergic system. Further, we will argue that as a result, care must be taken both in combining data from studies across cortical areas and species, and in choosing the best model systems to improve our understanding and support of human health.

## Introduction

Cholinergic modulation has been associated with a range of cognitive processes including learning and memory consolidation (Hasselmo and McGaughy, 2004), reward and addiction (Maskos et al., 2005; Kobayashi and Okada, 2007; Chubykin et al., 2013), plasticity (Bear and Singer, 1986; Weinberger, 2003), the sleep/wake cycle and arousal (Jasper and Tessier, 1971), perception (Kosovicheva et al., 2012; Boucart et al., 2015; Gratton et al., 2017), and attention (Everitt and Robbins, 1997; Sarter et al., 2005). In much of the literature exploring a role for cholinergic modulation, there is an implicit assumption that the various model systems used in these studies are equivalent. As such, the results of many experiments—spanning methodologies, species and cortical areas—are combined into unified models of function. This step assumes the existence of a canonical circuit for the cholinergic system in cortex. Even for cases of apparent functional equivalence, as will be discussed, the cholinergic system across model systems fails to exhibit anatomical equivalence. These anatomical differences make for unique cholinergic circuit compositions that may subserve similar processes across systems. With a primary focus on data from the rodent and primate cortex, we will describe anatomical differences in the cholinergic system both across and within species. We focus on these species not because we believe they are the only ones from which we can learn, but because they are the ones for which the published data are most complete. We argue that these existing data reveal that no canonical circuit for acetylcholine (ACh) exists in cortex. Further, they buttress the importance of consideration for species, cortical area and method of origin that inform functional models.

## Structure: Cholinergic Nuclei

In all mammalian species studied to date, the cell bodies of cholinergic projection neurons are located in subcortical nuclei of the brainstem and forebrain. Two major clusters of nuclei exist: the brainstem cholinergic system and the basal forebrain. The brainstem system is comprised of the pedunculopontine tegmental nucleus and the laterodorsal pontine tegmentum, which project to the basal ganglia, the thalamus and the basal forebrain. This review emphasizes the effects of ACh in cortex, and therefore focuses on the basal forebrain.

In primates, the basal forebrain is made up of the medial septal nucleus that projects to the hippocampus and entorhinal cortex, the diagonal band of Broca that projects to the hippocampus, entorhinal cortex and olfactory cortex, and the nucleus basalis/substantia innominata that provides ACh to the rest of cortex and to the amygdala (Mesulam et al., 1983). Neurons whose cell bodies reside in the basal forebrain are the only source of cortical ACh in adult primates (Mesulam et al., 1983). However, cell bodies immunoreactive for the synthetic enzyme choline acetyltransferase (ChAT) have been reported in the cortex of rats (Houser et al., 1983; Ichikawa and Hirata, 1986), cats (Avendaño et al., 1996), and fetal monkeys (Hendry et al., 1987; discussed below). In primates, the cholinergic projection neurons in the basal forebrain nuclei correspond to partially overlapping cell groups termed Ch1–Ch4. The medial septal nucleus corresponds to Ch1, the vertical limb of the diagonal band of Broca to Ch2, the horizontal limb of the diagonal band of Broca to Ch3, and the nucleus basalis/substantia innominata to Ch4. Similarly, the pedunculopontine tegmental nucleus and the laterodorsal pontine tegmentum of the brainstem projection system correspond to Ch5 and Ch6, respectively (Mesulam, 2004). There has been some debate regarding the extent to which the “Ch” nomenclature can extend to species beyond primates (i.e., to rodents and carnivores; Butcher and Semba, 1989), as the differentiation of cholinergic nuclei may be more subtle in other species (Gorry, 1963). Generally, however, the Ch1–Ch4 schema is considered applicable across species (Mesulam et al., 1983).

The “cholinergic” nuclei of the basal forebrain actually comprise a heterogeneous population of intermingled cholinergic, γ-aminobutyric acid (GABA)-ergic, and glutamatergic neurons (Mesulam et al., 1983; Gritti et al., 1997, 2006). Importantly, the proportions of the total neuronal population that each of these subpopulations represents are species-specific and differs between cholinergic nuclei within a single species. The methods used to define and quantify the cholinergic population within each nucleus have differed between studies, but the available data suggest a profound species difference between rodents and primates. Mesulam et al. (1983) report the proportion of cholinergic neurons in each of the Ch1–4 nuclei. The within-species variation is striking: 1% cholinergic in Ch3, through ~10% in Ch1, up to 70% and 90% in Ch2 and Ch4, respectively. The most comparable study in rodents does not report the proportion of cholinergic neurons for each nucleus individually, but the value of 5% given for the entire basal forebrain (Gritti et al., 2006) seems unlikely to arise from a population similar to that found in the primate, given that two large nuclei (Ch2 and Ch4) in the macaque each have a proportion many-fold higher than 5%.

The focus of this review is on cholinergic modulation in cortex. As such, the nucleus basalis/substantia innominata complex (NB/SI; the source of most cortical ACh, described above) will be the focus moving forward. The NB/SI in all species studied is heterogeneous. In humans, Ch4 alone contains approximately 220,000 neurons per hemisphere (Arendt et al., 1985). When one considers that Ch4 is one of two large nuclei in the basal forebrain (the other being Ch2) this would lead one to predict well over half a million neurons for the human basal forebrain total. For comparison, in rats, Ch1–4 together (i.e., the entire basal forebrain) contain 355,000 neurons per hemisphere (Gritti et al., 2006). Interestingly, Raghanti et al. (2011) report the number of *cholinergic* neurons in the human NB/SI to be between 200,000 and 230,000 per hemisphere, strikingly similar to the estimates for the *total* neuron number in human NB/SI. Accounting for individual variation, this estimate for cholinergic neurons may reflect a finding that in humans—as in macaques, but unlike rodents—90% of NB/SI neurons are cholinergic. Further species differences exist between primates regarding the number of cholinergic neurons in the NB/SI. The chimpanzee NB/SI is estimated to have 150,000–160,000 cholinergic neurons; estimates of that number for macaques range from approximately 90,000–120,000. Further, these estimates differ in tamarins, owl monkeys, capuchins, etc. (estimates are per hemisphere; Raghanti et al., 2011). These differences could be due to brain scaling (i.e., the number of neurons in the NB/SI increases as brain mass increases across animals). This, however, does not seem to be the case as the number of neurons in the NB/SI appears to increase at a slower rate than increases in brain mass across primates species (known as “hypo-scaling”; Raghanti et al., 2011).

Focusing specifically on cortically projecting neurons (and ignoring local circuit interneurons within the basal forebrain itself and neurons that project to other subcortical structures), two studies have combined retrograde tracing from the frontal lobe with visualization of ChAT immunoreactivity. In the rat, Gritti et al. (1997) have found that just under 20% of the cortically projecting basal forebrain neurons express ChAT, while in the macaque, the proportion is strikingly different at 96% (Mesulam et al., 1983). Proportions were similar for projections to the parietal lobe (rats: Gritti et al., 1997) and temporal lobe (primates: Mesulam et al., 1983). If the proportion of cholinergic and non-cholinergic neurons projecting to cortex from the basal forebrain differs between species this profoundly, it seems likely that the cortical modulation arising from activation of the basal forebrain nuclei may also be mechanistically different between species. It is interesting to note here that while there has been an overall hypo-scaling of subcortical areas with respect to cortical expansion over the evolutionary history of mammals (Raghanti et al., 2011; Collins et al., 2013), this increase from 20% to 96% ChAT-immunoreactive neurons suggests a massive functional hyper-scaling of the cholinergic projection to cortex in primates. Comparative studies are under way in our lab to determine what happened in the evolutionary history of the basal forebrain, and when; have rodents gained a large GABAergic population, or have primates lost one?

Cortical projections *to* the NB/SI of the macaque originate in the orbitofrontal cortex, temporal pole, prepyriform cortex, entorhinal cortex, inferotemporal cortex, insula and prefrontal cortex (Mesulam and Mufson, 1984). Subcortical projections to the NB/SI of the macaque originate in the medial hypothalamus, septal nuclei, nucleus accumbens-ventral pallidum and the amygdala (Price and Amaral, 1981; Mesulam and Mufson, 1984). NB/SI afferents in rats and cats are similar to primates (Haring and Wang, 1986; Irle and Markowitsch, 1986; Jones and Cuello, 1989). The NB/SI in rats and monkeys is also the target of extensive input from other neuromodulatory systems including the serotonergic, dopaminergic and noradrenergic systems (reviewed by Mesulam, 2004). Data are needed to describe the status of neuromodulatory inputs to the NB/SI in other species (such as the cat).

## Structure: Cholinergic Projections

Projection neurons of the Ch4 group innervate all of the neocortex, but the densities of these projections differ between and within cortical areas in all species studied to date. This is a notable characteristic of a cortical area because a denser network of cholinergic axons is likely to yield a higher density of cholinergic release sites, leading to more ACh being delivered. Unfortunately, the same type of data are not available for all animals studied, making comparison imperfect and interpretation difficult. There are, however, many examples of both species- and area-specific patterns of cholinergic innervation in cortex (examples briefly summarized in Table 1). In the human and the cat, primary visual area (V1) is less densely innervated by cholinergic axons compared to other primary sensory and motor areas (Mesulam et al., 1992; Avendaño et al., 1996). Cholinergic projections in the macaque also show clear differences between cortical areas, with macaque prefrontal area 4 (primary motor cortex, M1) being more densely innervated than prefrontal areas 9 and 32 (Raghanti et al., 2008). Further, Lysakowski et al. (1989) report temporoparietal association areas (e.g., areas 5 and 7) in the rat are least densely innervated compared to all other cortical regions examined. This study includes rat V1, which—as mentioned above—is the least densely innervated cortical area in both humans and cats, indicating an interesting species difference in relative V1 innervation. From an evolutionary perspective, this is notable as it appears to violate a reasonable assumption that animals phylogenetically closer to each other (e.g., humans and rats) would possess more similar characteristics than animals phylogenetically farther apart (e.g., humans and cats). As demonstrated in Table 1, data are needed to determine where along evolutionary development changes in cholinergic innervation patterns may have occurred. For instance, it could be hypothesized that the innervation pattern in rat cortex is in fact the outlier compared to carnivores and primates. Data from a wider range of species and sampling from a broader range of cortical areas will be needed to resolve questions of the evolutionary origin and significance of observed differences.

**Table 1:** Relative degree of cholinergic innervation density across species and area.

	Sensorimotor		Frontal		Primary auditory/visual		Parietal
Human	M1 (S1_n.d._)	>	32 > 9	>	A1 > V1	<	39 = 40
Chimpanzee	M1 (S1_n.d._)	>	32 = 9	>	A1?V1	?	n.d.
Macaque	M1?S1	>	9 > 32	>	A1?V1	?	n.d.
Cat	M1 = S1	>	n.d.	>	A1 > V1	?	n.d.
Rat	M1 = S1	=	4 = 8 = 10 = 11	>	A1 = V1	>	5 = 7

* > indicates innervation density is greater than, < indicates innervation density is less than, = indicates innervation density is similar, n.d. indicates areas for which no data are available (to our knowledge), ? indicates the relationship between regions is unknown. M1, primary motor area; S1, primary somatosensory area; A1, primary auditory area; V1, primary visual area; frontal and parietal areas correspond to Brodmann’s classification. Data for human: Mesulam et al. (1992), Raghanti et al. (2008); chimpanzee: Raghanti et al. (2008); macaque: Campbell et al. (1987), Lewis (1991); Raghanti et al. (2008); cat: Avendaño et al. (1996); rat: Eckenstein et al. (1988), Lysakowski et al. (1989)*.

Looking at clusters of cortical areas, rather than each area individually, distinct cholinergic innervation patterns are again evident between species. For example, unlike in the cat, rat primary sensory areas have innervation patterns that are more similar to each other, but differ from motor and frontal areas (Lysakowski et al., 1989; Avendaño et al., 1996). In the frontal cortex of humans, chimpanzees and macaques, cholinergic axons are most dense in area 4 relative to areas 9 and 32 (Raghanti et al., 2008). This differs from rat cortex, where frontal areas (such as areas 4, 8, 10 and 11) are more uniformly innervated (Lysakowski et al., 1989). Further, while primate species studied all show highest cholinergic density in area 4, area 9 is least densely innervated in humans, area 32 is least densely innervated in macaques, and both areas show comparable density in chimpanzees (Raghanti et al., 2008).

Laminar variations exist within cortical areas, and these patterns again differ between cortical areas and between species (examples briefly summarized in Table 2). For example, in rat V1 and primary auditory cortex A1, the densest cholinergic innervation is found in layer I and at the border between layers IV and V; the least dense innervation is found in layers II/III and VI (Lysakowski et al., 1989). In fact, this study reports as many as 13 different cholinergic laminar innervation patterns across rat cortex. The laminar pattern of cholinergic innervation in cat M1 shows densest innervation in layers I, II and superficial III, while the pattern in V1 is a densely innervated layer I and more uniform innervation in the remaining layers (Avendaño et al., 1996). In macaque A1, cholinergic axons are most dense in layers I, III, and IV (Campbell et al., 1987). However, while layer I is also found to be densely labeled in macaque frontal cortex, layers III and IV are least densely innervated (Lewis, 1991). In all frontal areas in primates, the superficial layers are more densely innervated than are the deeper layers. Interestingly, however, the morphological features of the axons differ between primate species, even where the innervation densities are similar. For example, cholinergic axons forming “clusters” are present in both human and chimpanzee frontal cortices, but not in macaque. These clusters have been previously described as possible indicators of cortical plasticity events (Mesulam et al., 1992). Thus, even across species with similar innervation, the exact morphologies of cholinergic axons may differ and contribute to functional circuit differences.

**Table 2:** Laminar variations in choline acetyltransferase fiber immunoreactivity across species and area.

	Layer I	Layer II	Layer III	Layer IV	Layer V	Layer VI
Macaque FC	++	+	−	−	−/+	−
Macaque A1	+	−	−/+	++	−	−
Rat A1	++	−	++	++*		−
Rat V1	++	−	−	++*		−
Cat V1	++	+	+	+	+	+
Cat M1	++	++	++/−	−	+/−	−

++ indicates densest innervation of cholinergic axons, + indicates moderate innervation, − indicates lightest innervation, *indicates the transition at the border between two layers, / indicates a transition within the superficial and deep portions of a layer. FC, frontal cortex; A1, primary auditory area; V1, primary visual area; M1, primary motor area. Data for rat: Lysakowski et al. (1989); cat: Avendaño et al. (1996); macaque: Campbell et al. (1987); Lewis (1991).

## Function: Lesions of the Basal Forebrain

The basal forebrain cholinergic system has long been implicated in modulating cognitive processes and brain states through its widespread cortical projections (described above). Early studies in rats used excitotoxic lesions of basal forebrain nuclei and were interpreted as indicating that the cholinergic system is particularly involved in learning and memory (Dubois et al., 1985; Hepler et al., 1985). However, excitotoxic lesions eliminate all cells types within the basal forebrain, and it is now apparent that cholinergic neurons in the rat represent a minority of basal forebrain neurons. Thus, it was realized that attributions of these effects to the cholinergic system had to be treated with caution. The development and use of the cholinergic-specific toxin 192 IgG-saporin (Wiley et al., 1991) revealed that, in rats, lesions that leave non-cholinergic cells intact result in a somewhat different pattern of cognitive deficits. For instance, performance in learning and memory tasks following cholinergic-specific lesions was either unimpaired or not as severely impaired compared to non-specific lesions (Berger-Sweeney et al., 1994; Wenk et al., 1994). This indicates that non-cholinergic cell types within the basal forebrain may contribute more to some processes, such as learning and memory, than do the cholinergic neurons.

While learning and memory appear to be less sensitive to cholinergic depletion, attentional processes are impaired following cholinergic-specific lesions in rats (reviewed by McGaughy et al., 2000). In one study by Waite et al. (1999), the multiple choice reaction time task was used to challenge sustained attention (also known as vigilance). Briefly, rats receive a food reward by selecting one out of a number of available ports, the correct port having been indicated by brief illumination of a light. Some manipulations to the task can increase the “attentional demand” such as changing the time between trials (inter-trial interval variability), and increasing the number of response ports (e.g., from five active ports to nine), making the task more difficult. During times of increased demand, task performance is impaired following cholinergic depletion in rats. This indicates that ACh may have a role in vigilance, especially when the task load is greater. Another study by Bucci et al. (1998) reports depletion of cholinergic innervation to posterior parietal cortex in rats (an area thought to be involved in attentive regulation) impairs the ability to increase processing capacity to meet task demands. Here, processing of a visual stimulus (a light) is assessed following manipulation of the predictive relationship between the light and an auditory stimulus (a tone) in relation to a food reward. Rats with depleted cholinergic innervation are impaired at appropriately shifting attention based on the predictive relationships between stimuli compared to control rats. In fact, many studies in rats have demonstrated impairments in vigilance tasks following cholinergic-selective depletion (Turchi and Sarter, 1997; McGaughy and Sarter, 1998; McGaughy et al., 2002). It is important to note here that attention is not a unitary phenomenon and these lesion studies explore a different process (vigilance) than do traditional studies of attention in humans and non-human primates (selective attention). We will discuss evidence for ACh’s role in selective attention below.

In mammals other than rats and mice, ME20.4 IgG-saporin is used to selectively eliminate cholinergic cells. In the marmoset, both excitotoxic and cholinergic-specific lesions to the nucleus basalis result in learning impairments (Ridley et al., 1986; Fine et al., 1997). In these studies, subjects were trained to perform a simple visual discrimination task in which the location of a food reward is signified by one of two objects; the object marking the reward location must be learned over a series of trials. Marmosets with both lesion types are impaired at this task relative to controls. Given that the primate NB/SI is 90% cholinergic, as discussed above, it is not surprising that the two lesion methodologies do not differ in this species as profoundly as they do in rats. In macaques, a study by Browning et al. (2009) assesses learning and memory following specific cholinergic depletion of the inferotemporal cortex. Here, macaques were trained to identify one of two objects in a complex scene. Identification of the correct object is rewarded with a food pellet (object-in-place scene learning). To assess memory, macaques are trained in a delayed nonmatch-to-sample task. In this task, a sample object is presented briefly then two objects appear, one being the sample object and one being a novel object. Subjects are rewarded with a food pellet for identifying the novel object. Cholinergic depletion does not result in impairments in either task (but see Turchi et al., 2005). Croxson et al. (2011) report no impairment in decision-making or in the object-in-place scene learning task described above, however, spatial working memory is impaired following cholinergic depletion of the prefrontal cortex. The latter was assessed using a spatial delayed response task, which requires subjects to remember the location of a food reward after a delay period. Thus, unlike in rodents, cholinergic depletion in macaques impairs spatial working memory. Macaques who have undergone non-selective excitotoxic lesions show impaired memory in the delayed nonmatch-to-sample task (described above as unimpaired by selective cholinotoxic lesions of cortex), however, the impairment follows combined lesions to the nucleus basalis, medial septal nuclei, and diagonal band of Broca (Aigner et al., 1991). Lesions to the nucleus basalis alone (or any of the cholinergic nuclei in isolation) do not result in any impairment.

As demonstrated, cholinergic depletion produces complex results. The resulting impairment (if any) will depend on a number of variables. These include the composition of the basal forebrain in the species under study (e.g., the difference between the proportions of cholinergic neurons in the primate NB/SI vs. the rat), the immunotoxin (e.g., excitotoxic vs. cholinotoxic), the location of the lesion (e.g., the combination of subcortical nuclei and/or the cortical area affected), and the task used to assess the many forms of cognition thought to rely, at least in part, on ACh. Given the considerable variation in study design and resulting impairments following cholinergic depletion, unifying or extending the results of lesion studies to yield descriptions or predictions that hold across species, brain areas, and tasks is challenging, at best.

## Structure: Cholinergic Interneurons in Cortex

A notable distinction between cholinergic systems across species is the presence or absence of cholinergic neurons within cortex. Many studies have reported an absence of cell bodies immunoreactive for ChAT in primate cortex (Hedreen et al., 1983; Mesulam et al., 1983; Raghanti et al., 2008). It is believed that ACh is instead delivered to primate cortex exclusively through projections from the basal forebrain. This, however, is not true for other species studied (cat and rat). Moreover, in animals where cholinergic cortical cell bodies have been reported, there are laminar- and area-specific densities and distributions. For example, in the rat, cholinergic neurons are found in all cortical layers except layer I, and are most dense in layers II and III (Houser et al., 1983; Ichikawa and Hirata, 1986; Mechawar et al., 2000). In the cat, cholinergic cell bodies are similarly most dense in layers II and III, but are also more numerous in motor and somatosensory cortices than in auditory and visual cortices (Avendaño et al., 1996). These neurons are non-pyramidal, and probably co-release GABA and perhaps peptide signaling molecules as well (Eckenstein and Thoenen, 1983; Parnavelas et al., 1986; Bayraktar et al., 1997).

## Structure: ACh Synthesis

The local regulation of ACh synthesis can influence the capacity for cholinergic signaling. Choline necessary for the synthesis of ACh is taken up by cholinergic neurons via high-affinity choline transporters (ChTs). As such, ChTs are the rate-limiting factor in ACh synthesis (Okuda and Haga, 2003). Regions of tissue with relatively high levels of ChTs likely have the ability to continue to synthesize ACh under high levels of “demand” (i.e., axonal activity) and will be able to release ACh in a more sustained fashion than areas with low levels of ChT expression. ChT expression, especially in combination with patterns of cholinergic innervation (described above), affords more finely tuned local regulation of ACh release. Such local differences could lead to marked variation in extracellular ACh levels, both tonically and in response to increased demand.

ChT distribution exhibits species and area specificity. While ChT immunoreactivity in fibers and puncta is consistently low across cortical areas in rats (Misawa et al., 2001), levels differ between cortical areas in macaques. For instance, the cingulate cortex exhibits higher levels of ChT immunoreactivity than do the frontal, parietal, temporal and occipital cortices (Kus et al., 2003). This suggests a higher capacity for sustained cholinergic signaling in cingulate cortex than in other cortical areas. Within each cortical area, in both species, there are also laminar differences in ChT expression, with deeper layers showing stronger immunoreactivity compared to superficial layers (Misawa et al., 2001; Kus et al., 2003). This may indicate a need for more sustained signaling in the layers of cortex that interact with subcortical structures than in the layers responsible for the majority of the feedforward and lateral interactions within cortex.

## Structure: Cholinergic Receptors

The cholinergic system transduces signals through two families of receptors: nicotinic and muscarinic, named for the exogenous ligands to which they are particularly responsive—nicotine and muscarine, respectively (reviewed by Dorostkar and Boehm, 2008). Nicotinic receptors are cation (ionotropic) channels, which mediate a fast depolarization of the receiving cellular membrane. As pentamers (i.e., containing five subunits), they can be characterized by their subunit composition. Heteropentameric nicotinic receptors contain two obligatory α subunits in addition to other subunits, usually β2 in cortex (Gotti et al., 2006; Dorostkar and Boehm, 2008; Albuquerque et al., 2009). The only homopentameric nicotinic receptors described so far in mammals comprise five α7 subunits.

Muscarinic receptors are metabotropic. They can act via direct channel coupling or through coupling to various intracellular second messenger systems. Five muscarinic receptor subtypes have been identified (m1–m5) and are characterized according to the class of G protein to which they are coupled (reviewed by Gilsbach and Hein, 2008). The m2 and m4 receptors are preferentially coupled to the Gi/o signaling pathway and correspond to the M2 pharmacological class (i.e., they are insensitive to the antagonist pirenzepine; MacIntosh, 1984). These receptors are typically expressed presynaptically and act as autoreceptors. The m1, m3 and m5 receptors are preferentially coupled to the Gq signaling pathway and correspond to the M1 pharmacological class, although sensitivity to pirenzepine varies within this class. These receptors are more often expressed postsynaptically. While the molecular signaling characteristics of these receptors are almost certainly the same in all species, their expression by neuron type differs.

## Structure: Cholinergic Receptor Expression by Inhibitory Neurons

Inhibitory interneurons in cortex (i.e., local circuit neurons that express GABA) exhibit considerable structural and functional diversity. One means of characterizing subpopulations of cortical inhibitory neurons is to classify them by their expression of molecular markers. The most commonly used classification schemes differ between species, as do the interneuron populations themselves. In macaques, the use of immunoreactivity for calcium-binding proteins is the most common scheme, but this may not apply well to other species. For example in rats, the calcium-binding protein parvalbumin (PV) is a marker for the fast-spiking physiological phenotype (Weiser et al., 1995; Sekirnjak et al., 1997), and is expressed by half of V1 inhibitory interneurons (Gonchar and Burkhalter, 1997). However, not all PV-immunoreactive (ir) neurons in the primate are fast-spiking (Härtig et al., 1999; Constantinople et al., 2009).

A comparative anatomy study by Disney and Reynolds (2014) reports species differences in cholinergic receptor expression by PV-ir neurons in V1. In humans, macaques, and guinea pigs, the majority of PV-ir neurons (76%–84%) expresses the m1 muscarinic receptor. In ferrets and rats, however, fewer than half of the PV-ir neurons express the m1 receptor. In rats, the low expression is observed in the primary somatosensory cortex (S1) as well as in V1. Further, a study in mice found m1 muscarinic receptor expression by inhibitory neurons, including PV-ir neurons, to be low or undetectable in cortex (Yamasaki et al., 2010). Thus, cholinergic receptor expression by PV-ir neurons differs across model systems, and the neuronal “class” defined by PV immunoreactivity may also differ. Other calcium-binding proteins, such as calbindin (CB) and calretinin (CR), are also used to classify inhibitory interneurons, particularly in primates. Disney and Aoki (2008) report 60% of CB-ir and 40% of CR-ir neurons express the m1 receptor in macaque V1. A smaller proportion of these populations expresses the m2 receptor at 23% of CB-ir neurons, 25% of CR-ir neurons, and 31% of PV-ir neurons. In rat V1, one *in vitro* study reports that ACh depolarizes low-threshold spiking cells via nicotinic receptors (Xiang et al., 1998). While this study does not report any molecular immunoreactivity for these neurons, previous work indicates low-threshold spiking neurons in rodent cortex correspond to neurons immunoreactive for CB (Kawaguchi and Kubota, 1993). In macaques, CB-ir neurons also express nicotinic receptors, as do PV-ir neurons, while expression by CR-ir neurons is rare (Disney et al., 2007).

Other molecular markers are often used to describe inhibitory populations in rodents. In a study by Kawaguchi (1997), *in vitro* recordings in rat frontal cortex show the extent to which GABAergic neurons immunoreactive for PV, somatostatin, vasoactive intestinal polypeptide (VIP) and cholecystokinin respond to cholinergic agonism. In the presence of carbachol (a cholinergic agonist) or muscarine (a muscarinic-specific agonist), VIP- and somatostatin-ir neurons are depolarized and cholecystokinin-ir neurons are either hyperpolarized or depolarized depending on their size. This finding indicates these populations likely express cholinergic receptors. Unlike in rat visual cortex where PV-ir neurons express the m1 receptor (albeit, a small population; discussed above), the PV-ir neurons in this study were unaffected by both carbachol and muscarine.

## Function: Cholinergic Modulation of Inhibition

In guinea pig, cat and macaque V1, muscarinic agonists can have a suppressive effect on the firing rate of neurons in cortex. At least some of this suppressive effect can be blocked by GABA antagonism (McCormick and Prince, 1986; Müller and Singer, 1989; Disney et al., 2012), indicating that ACh can induce the release of GABA. It is probable that this GABA release is mediated by the PV-ir, m1 receptor-expressing population in these species (discussed above). However, PV-ir neurons in rat visual cortex do not appear to mediate similar effects, as would be expected based on their lack of ACh receptor expression. *In vitro* studies in rat V1 show that ACh does not depolarize PV-ir neurons (Xiang et al., 1998; Gulledge et al., 2007). In contrast to rat, PV-ir neurons in mouse V1 are both excited and suppressed in response to optogenetic stimulation of basal forebrain cholinergic neurons (Alitto and Dan, 2013)—actions that were attributed to mixed activation of nicotinic and muscarinic receptor subtypes. As such, the capacity for ACh to induce the release of GABA from cortical interneurons may differ between species. This is particularly interesting in the context of the differing extent to which the NB/SI itself releases GABA in these species. Of note here is the apparent conflict between this study and the Yamasaki et al. (2010) finding described above (that m1 receptor expression by PV-ir neurons is low or undetectable). However, the latter study investigated cortex as whole, while the finding in Alitto and Dan (2013) is specific to V1.

## Structure: Cholinergic Receptor Expression by Excitatory Neurons

In macaques, receptor expression by excitatory neurons differs between cortical areas. In V1, the m1 receptor is expressed by over 60% of inhibitory neurons and by less than 10% of excitatory neurons (Disney et al., 2006). In visual area V2, m1 receptor expression by inhibitory neurons is similar to that in V1. However, muscarinic receptor expression by excitatory neurons differs sharply between V1 and V2, with at least double the proportion of excitatory neurons expressing muscarinic receptors in V2 compared to V1 (Disney et al., 2006). In fact, across the visual pathway—from V1, through V2, V3a, V4d, to MT—m1 receptor expression by inhibitory neurons remains roughly constant at 50%–60%, while m1 receptor expression by excitatory neurons differs with an increasing m1 receptor immunoreactive excitatory population moving “up” through the visual pathway (Disney et al., 2006, 2014; unpublished data). Thus, even within a species, cholinergic receptor expression by excitatory neurons differs between cortical areas.

The m1 receptor-expressing excitatory population also differs between species. As discussed above, only 10% of excitatory neurons in macaque V1 express the m1 receptor. In contrast, Gulledge et al. (2007) report between 25% and 95% (dependent upon layer) of excitatory neurons across rat cortex (medial prefrontal, somatosensory, and visual cortex) respond to ACh *in vitro*. Further, qualitative data indicate that excitatory neurons in V1 of ferrets and guinea pigs more frequently express the m1 receptor than do excitatory neurons in V1 of macaques or humans (Disney and Reynolds, 2014).

Regardless of neuron type (excitatory/inhibitory), cholinergic receptor expression in general is known to differ considerably across species. As demonstrated by *in vivo* recordings, 55% of cells in marmoset V1 respond to ACh (Roberts et al., 2005). This number in cats is between 85% and 90% (Müller and Singer, 1989; Stewart et al., 1999). With such variation not only in the proportion of ACh-responsive cells in an area, but also in the specific proportion of excitatory and inhibitory cells that express cholinergic receptors, effects of ACh observed in various cortical model systems may differ substantially.

## Structure: ACh Release

The instantaneous extracellular concentration of a given neuromodulator represents a critical feature of cortical circuits. This is because different ambient levels will yield activation of specific receptor subtypes based on receptor-specific affinity for the ligand, and—particularly for ACh—the rate of receptor desensitization. For example, muscarinic receptors m2 and m4 exhibit higher affinity for ACh than do the m1, m3, and m5 muscarinic receptors (Kuczewski et al., 2005). It can be hypothesized, then, that at lower levels of extracellular ACh, the m2 and m4 receptors will be more readily activated than will the m1, m3, or m5 receptors. Because the m2 and m4 receptors usually act as autoreceptors, their activation is likely to result in a different type of circuit regulation (reduced ACh release) than would the activation of the m1, m3 and/or m5 receptors. It is interesting that down-regulation of ligand release will likely be one of the earliest recruited cholinergic mechanisms in any circuit.

Similarly, the rate at which receptors desensitize will be related to extracellular ACh. For example, α7-containing nicotinic receptors become desensitized to ACh quickly, resulting in a decreased capacity for continuous cholinergic modulation. Non-α7-containing nicotinic receptors, however, desensitize more slowly, which likely results in prolonged cholinergic modulation. As such, modulatory signaling can differ in type and can range from more rapid to more prolonged, depending on the concentration-dependent recruitment of specific receptor subtypes. Altogether, the extracellular level of ACh will affect dynamics of receptor binding as well as the subtypes to which ACh will bind, and profound differences in these levels have been found across species.

In a review by Fitzgerald (2009), studies describing the concentration of ACh (as well as other neuromodulators) in awake macaque and rat prefrontal cortex were compared. These concentrations appear to be much higher in the macaque than in the rat. There are also differences in ACh levels within animals. For example, *in vivo* microdialysis in anesthetized rats shows baseline ACh levels are highest in the medial prefrontal cortex relative to visual cortex and somatosensory cortex (Fournier et al., 2004; but see Sarter and Bruno, 1997). Considering that a given ACh level will recruit specific receptor subtypes, which are themselves expressed differently by region and species, such variation in extracellular ACh levels may result in strikingly different cholinergic modulation of cortical circuits across and within species.

## Function: Effects of ACh Release in Cortical Circuits

It has been proposed that ACh facilitates attentive processes in sensory cortices. A suggested mechanism for this facilitation is the simultaneous enhancement of a sensory input and suppression of intrinsic cortical activation (Hasselmo and Giocomo, 2006). Many studies have described a population of nicotinic receptors in the input layer of a number of thalamic recipient cortical areas across species including rat, cat and primate (Clarke et al., 1984; London et al., 1985; Prusky and Cynader, 1986; Lavine et al., 1997; Disney et al., 2007; Eickhoff et al., 2007). These receptors were found to be located on terminals from thalamic nuclei innervating sensory (Prusky et al., 1987; Lavine et al., 1997; Disney et al., 2007), motor (Lavine et al., 1997), and association (Lavine et al., 1997; Lambe et al., 2003) cortical areas. Nicotinic receptors expressed presynaptically by thalamic terminals are thought to increase the gain of incoming sensory data (Disney et al., 2007; Kawai et al., 2007).

The thalamic enhancement/cortical suppression model of attention (Hasselmo and Giocomo, 2006) is based primarily on pharmacological studies in rodent sensory cortex *in vitro* (e.g., Hasselmo and Bower, 1992; Gil et al., 1997; Kimura et al., 1999; Hsieh et al., 2000). An interesting study from the point of view of species comparisons is that by Gil et al. (1997). In the somatosensory cortex *in vitro*, they report differences in the receptor population underlying cholinergic regulation of thalamocortical synapses between rats and mice. In both species, thalamocortical synaptic transmission is enhanced by nicotinic receptor agonists, and both thalamocortical and intracortical synapses are suppressed by muscarinic receptor activation. Interestingly, in this study, there was a difference between rats and mice in the nicotinic receptor subtype mediating the thalamic enhancement. Specifically, an α7 nicotinic receptor antagonist blocked the thalamocortical enhancement in rats, but not in mice, suggesting a species difference in receptor subtype expression at the thalamocortical synapse. This is of potential functional importance because the homomeric α7 receptor passes calcium with very high affinity (Vijayaraghavan et al., 1992; Séguéla et al., 1993), allowing nicotinic signaling to be directly coupled to intracellular plasticity mechanisms. Rats are the only species reported thus far to have a predominant α7-mediated nicotinic response reported at the thalamocortical synapse.

The suppression of intracortical pathways in these rodent studies has been attributed to a reduction in glutamate release through activation of m2 muscarinic receptors expressed on the axons of excitatory neurons (Hasselmo and McGaughy, 2004). However, another suppressive mechanism has been proposed for primates. In macaque V1, cholinergic suppression of visual responses was shown to be mediated by a strengthening of inhibition (Disney et al., 2012). Here, suppression by ACh is mediated by an increase in GABA release (as opposed to the reduction in glutamate release observed in rats). There is evidence that ACh also increases the strength of inhibition in the cat (Müller and Singer, 1989) and in the guinea pig (McCormick and Prince, 1986).

Interestingly, Soma et al. (2012) report a predominant enhancement by ACh (in contrast to the dominant suppression observed by Disney et al., 2012) mediated by muscarinic receptor activation, although both studies report both suppression and enhancement. The difference between these studies is not clear, but may be attributable to the concentration dependence of ACh effects or sampling bias in recording. Concentration-dependent effects will result from differing affinities of muscarinic receptor subtypes (discussed above; Kuczewski et al., 2005; Disney et al., 2012). Both of these studies were conducted under anesthesia and so basal levels of ACh in cortex may differ if the maintained depth of anesthesia differed. Further, differences in ejection barrel geometry and applied iontophoretic currents will yield different levels of delivered drug above that basal level. These factors can combine to activate different populations of receptors for subtly different experimental conditions. Neither study reported the proportion of interneurons in their recorded population, but given the anatomy of macaque V1, a recording bias towards excitatory neurons will also yield a higher proportion of suppression, and a recording bias towards interneurons would yield more apparent enhancement. It is important to note that across the population of neurons in V1, in all species studied, both enhancement and suppression are observed with cholinergic activation *in vivo*. What may differ between species and with methodology is the proportion of excitatory vs. suppressive effects, and perhaps the mechanism underlying the suppression, when observed (Sillito and Kemp, 1983; Sato et al., 1987; Müller and Singer, 1989; Murphy and Sillito, 1991; Zinke et al., 2006; Disney et al., 2007, 2012; Herrero et al., 2008; Soma et al., 2012).

In macaques and humans, there have been functional demonstrations of ACh enhancing sensory input relative to intrinsic cortical activity. One study in macaque V1 shows that administration of nicotine (a ligand for nicotinic receptors) improves contrast sensitivity (Disney et al., 2007) for V1 neurons. Here, anesthetized macaques were presented drift-grating stimuli of multiple contrasts with or without iontophoretic application of nicotine in V1. Physiological recordings reveal that in the presence of nicotine, cells in the input layer 4c produce reliable responses to lower-contrast stimuli, indicating that low-contrast detection is improved with nicotine. Similarly, in humans, cholinergic enhancement has been shown to increase signal detection. In a study by Boucart et al. (2015), participants engaged in a two alternative forced choice task in which they are shown two pictures of natural scenes, one of which contains an animal (the target). Participants must indicate which picture contains the target under varying levels of contrast. Before the task, participants were given either a placebo or the drug donepezil. Donepezil limits ACh degradation by inhibiting the ACh metabolizing enzyme acetylcholinesterase, and thereby enhances cholinergic transmission. In the presence of donepezil, signal detection of the target is facilitated. Of course, with systemic drug delivery such as this, there is no way to determine where in the brain the donepezil is acting to produce this behavioral effect. Further data would be needed to assign donepezil’s actions to the increased gain at the input to cortex.

Beyond the input layer, a decrease in receptive field size and a reduced spread of excitation have been proposed as measurable consequence of suppressing lateral cortical interaction. This is because the size of a receptive field center is thought to be largely determined by inputs arising from the thalamus, while the receptive field surround is provided by lateral and feedback connectivity within cortex (Angelucci and Bressloff, 2006). Silver et al. (2008) report cholinergic enhancement suppresses the spread of excitation in human V1. In this study, subjects passively viewed high-contrast/contrast-reversing checkerboards interspersed with a blank gray screen, while maintaining fixation on a central point. Positive blood-oxygen-level dependent (BOLD) responses to the checkerboard stimulus relative to baseline (the gray screen) were observed by functional magnetic resonance imaging (fMRI). Prior to fMRI sessions, subjects ingested either a placebo or donepezil (described above). Donepezil administration resulted in a positive BOLD response to stimuli that occupied less cortical surface area compared to placebo. This indicates cholinergic enhancement reduces the spatial spread of excitation. Similar effects have been observed in non-human primates. In a study by Roberts et al. (2005), length tuning of V1 neurons to bar stimuli was studied with and without iontophoretic application of ACh. In the presence of ACh, the neurons’ preferred stimulus length shifted toward shorter bars. This phenomenon was modeled as a reduction in the summation area of the neurons’ receptive fields, again consistent with a suppressive effect of cortical ACh release.

## ACh in Attention

The model proposed by Hasselmo and Giocomo (2006) may—at least partially—explain ACh’s role in attentive processing. Unfortunately, the current literature does not adequately distinguish between ACh’s direct involvement in attention vs. cholinergic modulation of vigilance (which is demonstrated in the lesion studies described above). It may be the case that vigilance—and therefore ACh—is a necessary, but not sufficient component of attention. In this case, the cholinergic modulation of vigilance would act as a condition upon which more selective or focal attention can be achieved.

As described earlier, the suggested mechanism for ACh’s facilitation of vigilance is the simultaneous enhancement of a sensory input and suppression of intrinsic cortical activation. To date, only one study has used a true selective attention task to investigate the local effects of ACh in the cortex of an awake, behaving primate (Herrero et al., 2008). Here, recordings were made in macaque V1 during performance of a cued contrast change detection task. In the task, macaques must maintain fixation at a central point and detect a luminance change at a cued location (target) while ignoring a luminance change in a non-cued location (distractor). The results show the expected increase in the firing rate of V1 neurons when a target is detected in their receptive fields, that is, when the target is “attended to.” Further, they demonstrate that V1 neurons show a greater attentional modulation during application of ACh. These effects were also shown to be the result of muscarinic receptor activation, as the muscarinic antagonist scopolamine reduced the attentional enhancement, while nicotinic antagonists had no effect on attentional modulation (although nicotinic antagonists had a generalized effect on spike rates). This study provides clear evidence that ACh is involved in some form of attentive processing that goes beyond vigilance in showing differences in the attend-to vs. attend-away conditions. The use of inter-hemispheric comparison for attend-to/attend-away, however, leaves open the question of whether ACh is sufficient for selective attentive effects, or whether another molecule supports cholinergic action at a finer scale in cortex. We are not aware of studies of cholinergic action on selective attention in species other than primates (human and non-human).

Other notable primate (both human and non-human) studies support a role of ACh in attention (e.g., Witte et al., 1997; Furey et al., 2008; Thiel and Fink, 2008); however, these studies cannot address the questions within the scope of this review, as they involve systematic administration of cholinergic drugs. In this case, drugs affect the entire body, leaving little ability to resolve different cholinergic effects across cortical areas, especially at the circuit level. Further, few species comparisons exist, as most of these studies are performed in human subjects.

## Conclusions

A thorough understanding of the differences that exist in cholinergic signaling across mammalian model systems commonly used in neuroscience is of particular importance for the cholinergic system. This system is traditionally considered broad in its impact throughout cortex because of the relatively small number of cholinergic neurons in the NB/SI, their large branching axonal arbors, and the evidence for volume transmission by ACh. Volume transmission is a diffuse signaling mode wherein a molecule is released from varicosities that are usually not apposed to a specialized receptive surface (demonstrated in both rats and primates; Umbriaco et al., 1994; Mrzljak et al., 1995; but see Turrini et al., 2001). Even synaptically-released ACh can spill over from the synapse and thus participate in volume transmission (Dani and Bertrand, 2007). When it is not confined to a synapse, ACh can diffuse through the extracellular space, activating nearby receptors. This capacity for diffuse signaling often leads to an implicit assumption that the cholinergic system lacks precision. However, variations in the anatomy across the arbor of individual cholinergic axons and differences in the receiving circuits in cortex may introduce regionally-specific responses to diffuse modulatory signals. Furthermore, differences in expression of acetylcholinesterase and the ChT, which serve to terminate cholinergic signaling, can also induce differences in the temporal precision of a cholinergic signal, whether or not that signal is spatially precise. The product of regional differences in anatomical features that determine the spatiotemporal properties of the cholinergic signal is the existence of unique neuromodulatory compartments across cortex (Coppola et al., 2016). Features such as patterns of axonal innervation to cortex, molecular diffusion, effectiveness of degradation and reuptake pathways, subcellular receptor localization, and patterns of receptor expression across local receiving circuits (among many others) can offer the capacity for local modulation of long-range communication between neurons.

A second common assumption is that the various model systems used to study ACh are equivalent and interchangeable, resulting in the possible existence of a canonical circuit for the cholinergic system in cortex. Here, we have discussed important differences in the structure and function of the cholinergic system both across and within species. Even for cases of apparent functional equivalence (such as the common observation of a relative enhancement of sensory input to cortex over intrinsic pathways in the presence of ACh), the cholinergic system across model systems fails to exhibit anatomical equivalence. The anatomical differences across species and areas make for unique cholinergic circuit compositions that may nonetheless yield similar computations across systems (i.e., computational equivalence in the absence of anatomical equivalence).

The differences between species should not be viewed as surprising; there is growing evidence from studies in invertebrates that modulatory systems are common sites of evolutionary elaboration (Grashow et al., 2009; Katz and Lillvis, 2014). That this phenomenon extends to vertebrates, specifically mammals, is both likely and—as we have shown here—already supported by the literature. At a conceptual level, anatomical differences may not matter given the evidence for computational or functional equivalence. However, if we are to use the data from studies of cholinergic structure and function to drive improvements in human health and treatment of disease, the precise mechanisms by which these computations are achieved matters, and thus is a critical consideration for future studies.

## Author Contributions

AAD conceived of the review topic and scope and assisted JJC in writing the review.

## Conflict of Interest Statement

The authors declare that the research was conducted in the absence of any commercial or financial relationships that could be construed as a potential conflict of interest. The reviewer IC and handling Editor declared their shared affiliation.
